# Supplementary material for: Pattern of COVID-19 in Sichuan province, China: A descriptive epidemiological analysis
Source: PLoS One. 2020 Nov 5;15(11):e0241470. doi: 10.1371/journal.pone.0241470 (PMC7644260; doi:10.1371/journal.pone.0241470)
Supplement: S1 File — (PDF) [file pone.0241470.s001.pdf]

Pattern of COVID-19 in Sichuan Province, China: a descriptive epidemiological analysis

Supplementary Material

Hongfei Song[1¶], Xiaoren Cao[2¶], Hua Ye[3¶], Li He[3¶], Guiyu Li[1], Tingjun Wan[1], Dong Wang[1], Yuqiao Liu[4], Zonghai Huang[3], Baixue Li[1], Li Wen[1], Yue Su[1], Cen Jiang[1*], Quansheng Feng[1*]

1) Data of 487 confirmed COVID-19 cases.

ID	Areas	S(onset)	Date of confirmed cases	Age	Gender	symptom
1[*]	Chengdu	Jan.11th	Jan.21st	34	M	Fever
2[*]	Chengdu	-	Jan.22nd	28	F	-
3[*]	Chengdu	Jan.18th	Jan.23rd	50	M	Fever, Cough, Dyspnea
4[*]	Chengdu	Jan.18th	Jan.23rd	48	M	Fever
5[*]	Chengdu	-	Jan.27th	59	F	-
6[*]	Chengdu	-	Jan.27th	38	M	-
7[#]	Chengdu	-	Jan.27th	37	M	-
8[*]	Chengdu	-	Jan.27th	49	M	-
9[*]	Chengdu	-	Jan.28th	31	M	-
10[*]	Chengdu	-	Jan.28th	59	F	-

11*	Chengdu	-	Jan.28th	19	F	-
12*	Chengdu	-	Jan.28th	25	M	-
13*	Chengdu	-	Jan.28th	32	M	-
14#	Chengdu	-	Jan.28th	50	F	-
15*	Chengdu	-	Jan.28th	19	F	-
16	Chengdu	-	Jan.28th	71	M	-
17*	Chengdu	-	Jan.28th	32	F	-
18*	Chengdu	-	Jan.29th	43	F	-
19*	Chengdu	-	Jan.29th	29	F	-
20#	Chengdu	-	Jan.29th	65	M	-
21	Chengdu	-	Jan.29th	45	F	-
22*	Chengdu	-	Jan.29th	47	F	-
23*	Chengdu	-	Jan.29th	68	F	-
24#	Chengdu	-	Jan.29th	33	F	-
25	Chengdu	-	Jan.29th	40	F	-
26*	Chengdu	-	Jan.29th	36	M	-
27*	Chengdu	-	Jan.29th	67	F	-
28#	Chengdu	-	Jan.29th	62	F	-
29*	Chengdu	-	Jan.29th	62	F	-
30*	Chengdu	-	Jan.29th	33	M	-
31#	Chengdu	-	Feb.1st	55	F	-
32*	Chengdu	-	Feb.2nd	33	F	-
33	Chengdu	-	Feb.2nd	76	F	-
34#	Chengdu	Jan.31st	Feb.2nd	22	F	Cough
35#	Chengdu	-	Feb.2nd	50	M	-
36#	Chengdu	Jan.28th	Feb.3rd	47	M	Cough
37#	Chengdu	Feb.2nd	Feb.3rd	47	M	Headache
38*	Chengdu	Feb.2nd	Feb.3rd	60	M	Fever
39#	Chengdu	Feb.2nd	Feb.3rd	65	M	Cough
40#	Chengdu	Jan.23rd	Feb.3rd	75	M	Fever
41#	Chengdu	Jan. 31st	Feb.3rd	49	M	Fever
42#	Chengdu	Jan.25th	Feb.3rd	30	F	Fever
43#	Chengdu	Jan.27th	Feb.3rd	78	F	Fever
44*	Chengdu	Jan.31st	Feb.3rd	47	M	Fever
45	Chengdu	Jan.30th	Feb.3rd	83	F	Fever
46*	Chengdu	-	Feb.4th	31	F	-
47*	Chengdu	Feb.2nd	Feb.4th	9	M	Cough
48*	Chengdu	Jan.31st	Feb.4th	47	M	Fever

49#	Chengdu	Feb.4th	Feb.4th	77	M	Fever
50*	Chengdu	-	Feb.4th	45	M	-
51	Chengdu	Jan.28th	Feb.5th	43	F	Diarrhea
52	Chengdu	Jan.28th	Feb.5th	64	F	Diarrhea
53	Chengdu	-	Feb.5th	55	M	Chills
54*	Chengdu	-	Feb.5th	51	M	-
55#	Chengdu	-	Feb.5th	47	F	-
56#	Chengdu	-	Feb.6th	49	M	-
57#	Chengdu	-	Feb.6th	46	F	-
58#	Chengdu	-	Feb.6th	17	M	-
59#	Chengdu	-	Feb.6th	33	M	-
60#	Chengdu	-	Feb.6th	53	M	-
61	Chengdu	-	Feb.7th	41	M	-
62#	Chengdu	-	Feb.7th	49	F	-
63#	Chengdu	-	Feb.7th	54	F	-
64#	Chengdu	-	Feb.7th	55	M	-
65*	Chengdu	-	Feb.7th	61	F	-
66*	Chengdu	-	Feb.7th	62	M	-
67#	Chengdu	-	Feb.7th	28	F	-
68*	Chengdu	Jan.23rd	Feb.8th	49	M	Fever
69*	Chengdu	-	Feb.8th	42	F	-
70*	Chengdu	Feb.4th	Feb.8th	81	M	Fever
71*	Chengdu	Feb.2nd	Feb.8th	23	F	Cough
72*	Chengdu	Jan.31st	Feb.8th	0.3	M	Cough
73*	Chengdu	Feb.3rd	Feb.8th	48	F	Cough
74*	Chengdu	-	Feb.8th	80	F	-
75	Chengdu	Feb.1st	Feb.8th	33	M	Sore throat
76	Chengdu	Feb.4th	Feb.8th	27	F	Sore throat, Cough
77#	Chengdu	-	Feb.9th	51	F	-
78#	Chengdu	Feb.1st	Feb.9th	49	F	Fever
79#	Chengdu	-	Feb.9th	67	F	-
80#	Chengdu	-	Feb.10th	29	F	-
81*	Chengdu	-	Feb.11th	28	F	-
82#	Chengdu	Jan.30th	Feb.12th	27	F	Cough
83#	Chengdu	Feb.6th	Feb.12th	87	M	Fever
84#	Chengdu	Feb.11th	Feb.12th	80	M	Stomach discomfort
85#	Chengdu	-	Feb.12th	56	F	-
86#	Chengdu	-	Feb.12th	51	M	-
87#	Chengdu	-	Feb.12th	54	F	-
88#	Chengdu	Feb.5th	Feb.13th	55	M	Fever
89	Chengdu	Feb.8th	Feb.13th	50	M	Cough

90#	Chengdu	-	Feb.13th	62	F	-
91#	Chengdu	-	Feb.13th	36	F	-
92#	Chengdu	Feb.5th	Feb.14th	88	F	Shortness of breath
93#	Chengdu	-	Feb.14th	49	F	-
94#	Chengdu	-	Feb.14th	85	F	-
95#	Chengdu	Feb.10th	Feb.14th	54	M	Fever
96	Chengdu	Feb.16th	Feb.18th	27	M	Fever
97*	Chengdu	-	Feb.20th	48	F	-
98#	Chengdu	-	Feb.20th	57	F	-
99	Chengdu	Mar.3rd	Mar.4th	30	F	Fever
100^	Chengdu	Feb.10th	Mar.17th	34	M	Cough, Sputum production, Runny
101^	Chengdu	Mar.19th	Mar.19th	55	F	Fever, Cough
102^	Chengdu	Mar.17th	Mar.20th	24	M	Fever, Chills, Shortness of breath
103^	Chengdu	Mar.21st	Mar.21st	25	M	Fever
104^	Chengdu	Mar.20th	Mar.23rd	21	M	Fever, Cough
105^	Chengdu	Mar.21st	Mar.23rd	37	M	Fever
106^	Chengdu	Mar.15th	Mar.24th	26	M	Dizziness, Nasal congestion, Sore throat
107^	Chengdu	Mar.24th	Mar.24th	24	F	Headache, Fever
108^	Chengdu	-	Mar.26th	62	F	-
109^	Chengdu	-	Mar.28th	25	F	-
110^	Chengdu	Mar.27th	Mar.28th	16	F	Fever, Headache
111^	Chengdu	Mar.31st	Mar.31st	22	F	Fever
112^	Chengdu	-	Mar.31st	27	F	-
113^	Chengdu	-	Apr.1st	0.2	M	-
114^	Chengdu	Mar.30th	Apr.1st	26	M	Fever
115^	Chengdu	Mar.10th	Apr.2nd	23	M	Headache, Fever
116^	Chengdu	-	Apr.3rd	23	M	-
117^	Chengdu	Apr.2nd	Apr.3rd	19	M	Nasal congestion, Runny
118^	Chengdu	Apr.3rd	Apr.4th	22	F	Fever
119^	Chengdu	-	Apr.5th	18	M	-
120^	Chengdu	-	Apr.6th	35	M	-
121*	Chengdu	-	Apr.17th	20	F	-
122*	Zigong	Jan.16th	Jan.24th	20	F	Cough, Sputum production
123*	Zigong	Jan.23rd	Jan.26th	34	M	Fever, Headache, Cough
124	Zigong	Jan.25th	Jan.27th	52	F	Sore throat, Fever
125	Zigong	Jan.26th	Jan.27th	49	F	Cough, Tightness in chest

126*	Zigong	Jan.26th	Jan.27th	40	M	Cough, Fever
127*	Zigong	Jan.25th	Jan.27th	67	F	Cough, Sputum production, Fever
128*	Zigong	Jan.25th	Jan.27th	42	F	Fever
129*	Zigong	Jan.28th	Jan.30th	39	F	Fever, Fatigue, Myalgia
130*	Zigong	Jan.29th	Jan.30th	48	F	Fever
131*	Panzhihua	Jan.28th	Jan.30th	38	F	Fever, Cough
132	Panzhihua	Jan.29th	Feb.1st	49	M	Fever, Cough
133	Panzhihua	Jan.29th	Feb.1st	48	F	Fever
134#	Panzhihua	Jan.29th	Feb.1st	50	F	Fever
135#	Panzhihua	Jan.29th	Feb.1st	24	M	Fever
136#	Panzhihua	Jan.29th	Feb.1st	46	M	Fever
137#	Panzhihua	Jan.29th	Feb.1st	18	M	Fever
138#	Panzhihua	Jan.30th	Feb.2nd	45	M	Fever, Cough
139#	Panzhihua	Jan.31st	Feb.2nd	54	M	Fever
140	Panzhihua	Jan.30th	Feb.3rd	47	M	Fever
141#	Panzhihua	Jan.30th	Feb.5th	22	M	Fever
142#	Panzhihua	Feb.3rd	Feb.8th	78	F	Fever, Cough
143#	Panzhihua	Feb.7th	Feb.9th	48	M	Fever
144#	Panzhihua	-	Feb.17th	79	F	-
145#	Panzhihua	-	Feb.17th	57	M	-
146#	Panzhihua	-	Feb.17th	61	M	-
147*	Luzhou	Jan.31st	Feb.1st	54	M	Fever, Cough
148*	Luzhou	Feb.1st	Feb.2nd	56	M	Fever, Cough
149*	Luzhou	-	Feb.3rd	53	F	-
150#	Luzhou	-	Feb.3rd	35	M	-
151	Luzhou	-	Feb.5th	28	F	-
152	Luzhou	-	Feb.5th	26	M	-

153#	Luzhou	-	Feb.6th	38	F	-
154	Luzhou	-	Feb.8th	52	M	-
155*	Luzhou	-	Feb.8th	10	M	-
156*	Luzhou	-	Feb.8th	47	F	-
157*	Luzhou	Feb.9th	Feb.9th	54	F	Sore throat, Diarrhea
158#	Luzhou	Feb.10th	Feb.10th	37	F	Sore throat, Sputum production
159#	Luzhou	Feb.10th	Feb.11th	49	M	Cough, Tightness in chest
160#	Luzhou	Feb.11th	Feb.18th	50	F	Headache, Cough, Sputum production
161*	Luzhou	Jan.23rd	Feb.19th	51	F	Cough, Sputum production
162*	Luzhou	-	Feb.19th	50	M	-
163*	Luzhou	-	Feb.19th	23	M	-
164#	Luzhou	Feb.6th	Feb.19th	28	M	Sore throat, Diarrhea, Chills
165*	Deyang	Jan.22nd	Jan.24th	39	M	Fever, Cough
166*	Deyang	-	Jan.28th	47	F	-
167*	Deyang	-	Jan.29th	53	M	-
168#	Deyang	-	Jan.30th	74	M	-
169#	Deyang	-	Jan.30th	68	F	-
170#	Deyang	-	Jan.30th	47	M	-
171#	Deyang	-	Jan.30th	44	F	-
172	Deyang	-	Feb.1st	45	M	-
173#	Deyang	-	Feb.1st	66	F	-
174*	Deyang	-	Feb.1st	29	F	-
175#	Deyang	Jan.30th	Feb.3rd	44	M	Fever
176*	Deyang	Feb.1st	Feb.3rd	70	F	Fever
177*	Deyang	Feb.7th	Feb.7th	31	M	Fever
178#	Deyang	Feb.2nd	Feb.8th	45	M	Cough

179*	Deyang	-	Feb.8th	5	M	-
180*	Deyang	-	Feb.8th	49	F	-
181#	Deyang	-	Feb.12th	37	F	-
182#	Deyang	-	Feb.20th	0.1	M	-
183	Mianyang	Jan.20th	Jan.22nd	37	M	Fever, Chills, Myalgia
184	Mianyang	Jan.20th	Jan.22nd	19	M	Fatigue, Sore throat, Myalgia
185	Mianyang	Jan.19th	Jan.26th	19	M	Dizziness, Fever, Cough
186	Mianyang	Jan.23th	Jan.26th	43	M	Chills, Fatigue, Headache, Myalgia and arthral
187	Mianyang	Jan.24th	Jan.26th	35	F	Fever, Myalgia
188	Mianyang	Jan.20th	Jan.26th	49	M	Fever, Cough, Sputum production
189	Mianyang	Jan.25th	Jan.26th	34	M	Fever, Cough, Sore throat, Myalgia
190	Mianyang	Jan.21st	Jan.27th	34	F	Nasal congestion, Runny, Fever, Sputum production
191*	Mianyang	Jan.19th	Jan.29th	51	F	Cough, Sputum production
192#	Mianyang	Jan.27th	Jan.30th	26	M	Cough
193*	Mianyang	Jan.28th	Jan.30th	25	M	Headache, Fever
194*	Mianyang	Jan.28th	Jan.31st	37	F	Cough
195*	Mianyang	Jan.25th	Feb.1st	73	F	Fever, Sore throat, Fatigue
196*	Mianyang	Jan.24th	Feb.1st	75	M	Fever, Sore throat
197*	Mianyang	Jan.26th	Feb.2nd	42	M	Fever, Cough
198*	Mianyang	Jan.31st	Feb.2nd	18	F	Fever
199	Mianyang	Jan.28th	Feb.2nd	36	M	Headache, Fever
200#	Mianyang	Jan.25th	Feb.5th	33	M	Fever, Fatigue
201*	Mianyang	Feb.5th	Feb.6th	7	M	Fever
202#	Mianyang	Feb.1st	Feb.7th	57	F	Fever, Chills
203#	Mianyang	Feb.3rd	Feb.9th	19	F	Cough, Nasal congestion, Sneezing, Tightness in chest, Dyspnea

204*	Mianyang	Feb.10th	Feb.11th	57	F	Cough
205*	Guangyuan	Jan.26th	Jan.28th	42	M	Cough, Shortness of breath, Stomach discomfort, Fatigue, Diarrhea
206*	Guangyuan	-	Jan.29th	37	F	-
207*	Guangyuan	-	Jan.29th	13	M	-
208*	Guangyuan	-	Jan.31st	46	F	-
209*	Guangyuan	-	Jan.31st	59	M	-
210*	Guangyuan	-	Feb.3rd	51	M	-
211#	Suining	Jan.14th	Jan.23rd	30	F	Fever, Sputum production
212*	Suining	Jan.22nd	Jan.25th	30	F	Cough, Fever, Fatigue
213	Suining	-	Jan.27th	24	F	-
214	Suining	-	Jan.29th	34	M	-
215	Suining	Jan.30th	Jan.30th	29	F	Headache, Sore throat,
216*	Suining	-	Feb.8th	35	M	-
217*	Suining	-	Feb.8th	38	F	-
218	Suining	Jan.29th	Feb.9th	29	F	Fever
219	Suining	-	Feb.9th	4	M	-
220#	Neijiang	Jan.23th	Jan.24th	44	F	Shortness of breath, Stomach discomfort, Fatigue, Diarrhea
221*	Neijiang	-	Jan.27th	47	M	-
222*	Neijiang	-	Jan.27th	16	M	-
223	Neijiang	Jan.25th	Jan.28th	31	M	Fever
224	Neijiang	Jan.26th	Jan.30th	52	M	Fever
225	Neijiang	Jan.29th	Jan.30th	36	M	Fever
226	Neijiang	Jan.30th	Jan.31st	37	M	Fever
227	Neijiang	Jan.30th	Jan.31st	21	M	Fever
228	Neijiang	Jan.30th	Jan.31st	36	M	Fever
229	Neijiang	Jan.30th	Jan.31st	29	F	Fever
230*	Neijiang	Jan.29th	Feb.2nd	46	M	Chills, Fever
231# &	Neijiang	-	Feb.3rd	36	M	-

232# &	Neijiang	-	Feb.3rd	34	F	-
233*	Neijiang	-	Feb.3rd	49	F	-
234*	Neijiang	Feb.4th	Feb.5th	56	F	Fever, Chills, Headache, Fatigue
235*	Neijiang	-	Feb.6th	45	M	-
236	Neijiang	Feb.3rd	Feb.10th	51	M	Fever
237#	Neijiang	-	Feb.11th	65	M	-
238#	Neijiang	-	Feb.11th	57	F	-
239#	Neijiang	Feb.11th	Feb.12th	22	F	Fever, Cough
240#	Neijiang	-	Feb.14th	47	F	-
241*	Leshan	Jan.25th	Jan.27th	32	M	Cough, Sputum production
242#	Leshan	-	Feb.6th	49	F	-
243	Nanchong	Jan.25th	Jan.27th	32	F	Fever
244	Nanchong	Jan.24th	Jan.27th	25	M	Fever
245	Nanchong	Jan.26th	Jan.28th	55	M	Fever
246	Nanchong	Jan.23th	Jan.29th	51	F	Fever
247	Nanchong	Jan.26th	Jan.29th	46	M	Fever
248	Nanchong	Jan.26th	Jan.29th	23	M	Fever
249	Nanchong	Jan.26th	Jan.29th	30	F	Fever
250	Nanchong	Jan.27th	Jan.30th	45	M	Fever
251	Nanchong	Jan.28th	Jan.30th	61	F	Fever
252	Nanchong	Jan.29th	Jan.31st	59	M	Fever
253	Nanchong	Jan.29th	Jan.31st	50	F	Fever
254	Nanchong	Jan.29th	Jan.31st	42	M	Fever
255	Nanchong	Jan.29th	Jan.31st	35	M	Fever
256	Nanchong	Jan.30th	Jan.31st	26	F	Fever
257	Nanchong	Jan.28th	Feb.1st	54	F	Fever
258	Nanchong	Jan.29th	Feb.1st	43	F	Fever
259	Nanchong	Jan.29th	Feb.1st	77	M	Fever
260	Nanchong	Jan.30th	Feb.1st	31	M	Fever
261	Nanchong	Jan.30th	Feb.1st	40	F	Fever
262	Nanchong	Jan.30th	Feb.1st	47	M	Fever
263	Nanchong	Jan.30th	Feb.1st	60	M	Fever
264	Nanchong	Jan.30th	Feb.2nd	9	M	Fever
265	Nanchong	Jan.30th	Feb.2nd	38	F	Fever

266* &	Nanchong	-	Feb.2nd	43	M	-
267	Nanchong	Jan.31st	Feb.3rd	46	F	Fever
268*	Nanchong	Feb.1st	Feb.4th	30	F	Fever
269	Nanchong	-	Feb.5th	67	F	-
270#	Nanchong	-	Feb.6th	35	M	-
271	Nanchong	Feb.3rd	Feb.7th	33	M	Fever
272#	Nanchong	Feb.2nd	Feb.8th	47	F	Fever
273#	Nanchong	-	Feb.9th	46	F	-
274	Nanchong	-	Feb.10th	48	F	-
275	Nanchong	-	Feb.12th	56	M	-
276# &	Nanchong	-	Feb.17th	56	M	-
277	Nanchong	-	Feb.17th	46	M	-
278*	Nanchong	Feb.13th	Feb.17th	37	M	Fatigue
279#	Nanchong	-	Feb.23rd	37	F	-
280*	Yibin	Jan.24th	Jan.26th	32	M	Fever, Fatigue, Cough, Dyspnea
281*	Yibin	Jan.23rd	Jan.28th	42	M	Chills, Fever
282*	Yibin	Jan.26th	Jan.29th	33	F	Fever, Fatigue
283*	Yibin	Jan.27th	Jan.29th	30	F	Fever, Fatigue
284*	Yibin	Jan.27th	Jan.29th	20	M	Fever
285	Yibin	Jan.28th	Jan.30th	44	M	Fever
286*	Yibin	Jan.28th	Jan.30th	22	M	Fever, Fatigue
287*	Yibin	-	Feb.5th	38	M	-
288*	Yibin	Feb.1st	Feb.6th	34	F	Cough
289*	Yibin	Feb.8th	Feb.11th	64	F	Cough, Sputum production
290*	Yibin	Feb.5th	Feb.12th	41	F	Cough, Tightness in chest
291*	Yibin	Feb.8th	Feb.17th	10	M	Fever
292*	Guangan	Jan.16th	Jan.21st	57	M	Fever, Cough
293*	Guangan	Jan.21st	Jan.24th	30	M	Fever

294*	Guangan	Jan.24th	Jan.27th	52	F	Fever
295*	Guangan	Jan.27th	Jan.29th	69	M	Fever
296*	Guangan	Jan.25th	Jan.29th	33	M	Fever
297	Guangan	Jan.25th	Jan.30th	48	F	Fever
298	Guangan	Jan.25th	Jan.30th	30	F	Fever
299	Guangan	Jan.29th	Jan.30th	50	F	Fever
300	Guangan	Jan.29th	Jan.30th	43	F	Fever
301	Guangan	Jan.30th	Jan.31st	35	M	Fever
302	Guangan	Jan.30th	Jan.31st	57	F	Cough, Shortness of breath
303	Guangan	Jan.29th	Jan.31st	46	M	Fever
304*	Guangan	Jan.31st	Feb.1st	41	F	Fever
305*	Guangan	Jan.30th	Feb.1st	64	F	Cough, Shortness of breath
306	Guangan	Jan.30th	Feb.1st	55	F	Fever
307	Guangan	Jan.30th	Feb.1st	31	M	Fever
308*	Guangan	Jan.29th	Feb.2nd	43	M	Fever
309#	Guangan	Jan.28th	Feb.3rd	48	F	Fever
310#	Guangan	Jan.28th	Feb.3rd	74	F	Fever
311	Guangan	Feb.1st	Feb.3rd	32	F	Fever
312	Guangan	Feb.5th	Feb.6th	49	F	Cough
313	Guangan	-	Feb.6th	45	M	-
314	Guangan	-	Feb.6th	21	M	-
315	Guangan	Jan.31st	Feb.7th	57	M	Cough
316#	Guangan	Feb.8th	Feb.10th	24	F	Fever
317	Guangan	Feb.8th	Feb.10th	55	M	Fever
318*	Dazhou	Jan.19th	Jan.28th	38	F	Fever, Cough, Runny
319*	Dazhou	-	Jan.29th	49	M	-
320*	Dazhou	-	Jan.29th	56	F	-
321*	Dazhou	-	Jan.30th	38	F	-
322*	Dazhou	-	Jan.30th	34	M	-
323	Dazhou	Jan.22nd	Jan.31st	45	M	Chills, Fever
324	Dazhou	Jan.31st	Feb.2nd	55	M	Chills, Fever

325	Dazhou	Jan.26th	Feb.2nd	46	F	Chills, Fever
326	Dazhou	Jan.27th	Feb.3rd	39	M	Chills, Fever
327	Dazhou	Feb.1st	Feb.3rd	49	F	Chills, Fever
328*	Dazhou	-	Feb.4th	43	M	-
329*	Dazhou	Jan.31st	Feb.4th	33	F	Fever, Cough
330*	Dazhou	Feb.2nd	Feb.4th	32	M	Fever, Cough
331	Dazhou	Feb.4th	Feb.4th	65	M	Cough
332*	Dazhou	Feb.1st	Feb.5th	56	F	Fever, Cough, Runny
333	Dazhou	Feb.2nd	Feb.5th	62	F	Sputum production, Fever
334	Dazhou	Feb.2nd	Feb.5th	65	F	Sputum production, Fever
335	Dazhou	Jan.30th	Feb.6th	45	M	Sputum production, Fever
336	Dazhou	Feb.4th	Feb.6th	35	F	Sputum production, Fever
337	Dazhou	Jan.26th	Feb.6th	65	F	Sputum production, Fever
338	Dazhou	Feb.5th	Feb.7th	34	M	Cough
339*	Dazhou	Jan.28th	Feb.7th	33	M	Fever, Fatigue, Chills
340	Dazhou	Feb.5th	Feb.9th	42	F	Cough
341	Dazhou	Feb.3rd	Feb.9th	26	M	Cough, Fatigue
342	Dazhou	Feb.6th	Feb.9th	53	F	Cough
343	Dazhou	Feb.8th	Feb.10th	30	F	Cough
344	Dazhou	Feb.8th	Feb.10th	52	F	Cough, Shortness of breath
345*	Dazhou	Feb.3rd	Feb.11th	46	M	Cough
346	Dazhou	Feb.5th	Feb.11th	63	F	Cough
347	Dazhou	Feb.7th	Feb.11th	49	M	Fever, Cough
348	Dazhou	Feb.9th	Feb.12th	46	F	Cough, Diarrhea
349*	Dazhou	-	Feb.12th	48	F	-
350*	Dazhou	-	Feb.12th	49	F	-
351#	Dazhou	-	Feb.16th	82	F	-
352#	Dazhou	-	Feb.16th	30	F	-
353	Dazhou	Feb.15th	Feb.17th	30	M	Fever, Cough
354#	Dazhou	-	Feb.18th	58	F	-
355#	Dazhou	-	Feb.20th	28	F	-
356	Dazhou	-	Feb.24th	65	F	-
357*	Bazhong	Jan.26th	Jan.30th	43	M	Fever, Cough, Sore throat, Fatigue
358*	Bazhong	Jan.26th	Jan.30th	42	F	Fever

359*	Bazhong	Jan.26th	Jan.30th	52	F	Fever
360* &	Bazhong	-	Jan.30th	19	M	-
361*	Bazhong	Jan.29th	Jan.30th	48	F	Fever
362*	Bazhong	Jan.28th	Jan.31st	50	M	Fatigue, Cough
363*	Bazhong	-	Jan.31st	26	M	-
364*	Bazhong	-	Jan.31st	46	M	-
365*	Bazhong	Jan.28th	Feb.2nd	38	M	Myalgia and arthral , Fever
366*	Bazhong	Jan.31st	Feb.2nd	34	M	Fever
367* &	Bazhong	-	Feb.2nd	34	M	-
368*	Bazhong	Jan.28th	Feb.2nd	47	F	Fever
369	Bazhong	-	Feb.4th	39	M	-
370*	Bazhong	-	Feb.4th	45	M	-
371*	Bazhong	Feb.1st	Feb.4th	11	M	Fever
372*	Bazhong	-	Feb.5th	28	F	-
373*	Bazhong	-	Feb.5th	60	F	-
374*	Bazhong	-	Feb.5th	4	M	-
375*	Bazhong	-	Feb.5th	2	M	-
376*	Bazhong	-	Feb.6th	19	M	-
377#	Bazhong	-	Feb.7th	53	F	-
378	Bazhong	-	Feb.9th	54	F	-
379#	Bazhong	-	Feb.11th	45	F	-
380#	Bazhong	-	Feb.13th	18	F	-
381*	Yaan	Jan.22nd	Jan.24th	28	M	Fever, Cough, Dyspnea
382*	Yaan	Jan.27th	Jan.30th	38	M	Fever, Cough
383	Yaan	Jan.29th	Jan.31st	64	M	Fever, Cough, Sore throat, Fatigue

384*	Yaan	Jan.27th	Jan.31st	69	M	Cough, Sputum production
385*	Meishan	Jan.23rd	Jan.26th	43	M	Fever, Cough
386*	Meishan	Jan.27th	Jan.29th	52	M	Fever, Cough
387	Meishan	Jan.30th	Feb.2nd	29	M	Cough, Sputum production, Fever
388	Meishan	Jan.27th	Feb.3rd	57	M	Fatigue, Fever
389$^{\#}$	Meishan	Jan.25th	Feb.3rd	50	F	Fatigue
390	Meishan	Jan.26th	Feb.7th	56	F	Cough, Fever
391	Meishan	Jan.26th	Feb.8th	63	M	Cough
392*	Meishan	Feb.1st	Feb.11th	49	F	Cough
393*	Ziyang	Jan.23rd	Jan.27th	21	M	Fever, Cough, Fatigue
394*	Ziyang	Jan.24th	Jan.27th	29	M	Cough
395*	Ziyang	Jan.27th	Feb.8th	41	M	Cough, Sputum production
396*	Aba	Jan.28th	Jan.29th	26	F	Fever
397*	Ganzi	Jan.23rd	Jan.25th	25	F	Fever
398*	Ganzi	Jan.23rd	Jan.25th	33	M	Fever
399	Ganzi	Jan.24th	Jan.27th	51	M	Fever
400*	Ganzi	Jan.27th	Jan.29th	49	F	Fever, Headache, Fatigue
401*	Ganzi	-	Jan.29th	49	M	-
402	Ganzi	-	Feb.2nd	51	M	-
403	Ganzi	Jan.24th	Feb.3rd	51	M	Myalgia, Chills , Cough
404	Ganzi	Jan.25th	Feb.3rd	36	M	Cough, Dyspnea
405$^{\#}$	Ganzi	-	Feb.4th	49	M	-
406$^{*\ \&}$	Ganzi	Feb.3rd	Feb.4th	28	F	Cough, Sore throat, Fever
407$^{\#}$	Ganzi	-	Feb.5th	66	M	-
408$^{\#}$	Ganzi	-	Feb.5th	48	M	-
409$^{\#}$	Ganzi	-	Feb.6th	13	M	-
410$^{\#}$	Ganzi	-	Feb.6th	55	F	-

411#	Ganzi	-	Feb.6th	13	M	-
412	Ganzi	Feb.2nd	Feb.6th	56	M	Nausea and stomach discomfort
413	Ganzi	Feb.4th	Feb.6th	50	M	Chills, Cough
414	Ganzi	Jan.25th	Feb.9th	54	M	Cough, Fever
415#	Ganzi	-	Feb.9th	37	F	-
416#	Ganzi	-	Feb.9th	47	M	-
417#	Ganzi	-	Feb.9th	28	F	-
418#	Ganzi	-	Feb.9th	33	F	-
419	Ganzi	-	Feb.9th	38	M	-
420#	Ganzi	-	Feb.10th	22	F	-
421#	Ganzi	-	Feb.10th	25	F	-
422#	Ganzi	-	Feb.10th	65	F	-
423#	Ganzi	-	Feb.10th	46	F	-
424#	Ganzi	-	Feb.11th	20	F	-
425#	Ganzi	-	Feb.11th	72	F	-
426#	Ganzi	-	Feb.11th	18	M	-
427#	Ganzi	-	Feb.11th	71	M	-
428#	Ganzi	-	Feb.11th	71	M	-
429#	Ganzi	-	Feb.11th	28	F	-
430#	Ganzi	-	Feb.11th	3	M	-
431#	Ganzi	-	Feb.11th	12	M	-
432#	Ganzi	-	Feb.11th	57	M	-
433	Ganzi	-	Feb.12th	49	F	-
434	Ganzi	Feb.6th	Feb.13th	39	F	Cough, Headache, Fatigue
435#	Ganzi	Feb.12th	Feb.13th	48	F	Tightness in chest, Shortness of breath
436#	Ganzi	-	Feb.13th	47	M	-

437#	Ganzi	-	Feb.13th	24	F	-
438#	Ganzi	-	Feb.13th	76	M	-
439	Ganzi	Feb.11th	Feb.13th	55	F	Myalgia, Fatigue, Cough
440#	Ganzi	-	Feb.14th	21	F	-
441	Ganzi	-	Feb.15th	30	M	-
442#	Ganzi	-	Feb.15th	43	M	-
443#	Ganzi	-	Feb.15th	54	F	-
444	Ganzi	-	Feb.15th	65	M	-
445	Ganzi	-	Feb.15th	77	F	-
446	Ganzi	-	Feb.15th	6	F	-
447	Ganzi	-	Feb.16th	41	M	-
448	Ganzi	-	Feb.16th	51	M	-
449	Ganzi	-	Feb.16th	56	M	-
450	Ganzi	-	Feb.16th	76	F	-
451	Ganzi	-	Feb.16th	54	F	-
452#	Ganzi	-	Feb.16th	20	F	-
453#	Ganzi	-	Feb.16th	53	M	-
454	Ganzi	-	Feb.17th	39	M	-
455	Ganzi	-	Feb.17th	54	M	-
456	Ganzi	-	Feb.17th	36	M	-
457	Ganzi	-	Feb.17th	41	M	-
458	Ganzi	-	Feb.17th	36	F	-
459	Ganzi	-	Feb.18th	18	M	-
460	Ganzi	-	Feb.18th	53	F	-
461	Ganzi	-	Feb.18th	43	F	-
462	Ganzi	-	Feb.19th	70	M	-
463	Ganzi	-	Feb.19th	54	M	-
464#	Ganzi	-	Feb.20th	54	F	-
465#	Ganzi	-	Feb.21st	47	M	-
466	Ganzi	-	Feb.25th	66	F	-
467	Ganzi	-	Feb.25th	62	M	-
468	Ganzi	-	Feb.26th	28	F	-
469	Ganzi	-	Feb.26th	38	F	-
470	Ganzi	-	Feb.26th	27	F	-
471	Ganzi	-	Feb.27th	46	M	-
472	Ganzi	-	Feb.27th	18	F	-
473	Ganzi	-	Feb.27th	46	M	-
474	Ganzi	-	Feb.27th	30	M	-

475*	Liangshan	Jan.23rd	Jan.26th	28	F	Fever, Cough
476*	Liangshan	-	Jan.27th	32	M	-
477*	Liangshan	-	Jan.30th	43	M	-
478*	Liangshan	-	Jan.31st	23	F	-
479*	Liangshan	-	Jan.31st	56	M	-
480*	Liangshan	-	Feb.2nd	29	F	-
481*	Liangshan	-	Feb.4th	64	F	-
482	Liangshan	-	Feb.7th	53	M	-
483	Liangshan	-	Feb.7th	23	M	-
484*	Liangshan	Feb.9th	Feb.9th	37	M	Fever, Cough
485*	Liangshan	-	Feb.14th	32	M	-
486#	Liangshan	-	Feb.14th	66	M	-
487#	Liangshan	-	Feb.15th	64	F	-

* COVID-19 cases with a history of living in or visiting Wuhan.
# COVID-19 cases with a history of close contact.
^ imported cases.
& cases with clinical symptoms, but no specific symptoms are specified
Unknown are not marked at all.
- Not available
