# Supplementary material for: Pattern of COVID-19 in Sichuan province, China: A descriptive epidemiological analysis
Source: PLoS One. 2020 Nov 5;15(11):e0241470. doi: 10.1371/journal.pone.0241470 (PMC7644260; doi:10.1371/journal.pone.0241470)
Supplement: Attachment — Submitted filename: Response to Reviewers.docx [file pone.0241470.s002.docx]

Dear Dr. Samy and Reviewers:

Thank you for your kind help in handling our manuscript ( #PONE-D-20-09556, Pattern of COVID-19 in Sichuan Province, China: a descriptive epidemiological analysis) to PLOS ONE.

The comments of you and reviewer were highly insightful and enabled us to greatly improve the quality of our manuscript. We have performed the suggested analyses and revised the manuscript according to your editorial comments and the reviewers’ comments. We marked all new changes using "Track changes while editing" in the revised text. In details, we address the issues in the comments point-by-point as follows.

We hope that now, after the changes we have carried out in the light of the reviewers’ comments, our revised manuscript is in an appropriate format for publication in PLOS ONE. We look forward to hearing from you.

Thank you and best regards.

Yours sincerely,

Corresponding author: QuanshengFeng: School of Basic Medical Sciences, Chengdu University of Traditional Chinese Medicine,No.1166 Liutai Avenue,Wenjiang District, Chengdu, Sichuan, China 611137;

E-mail:fengqs118@163.com.

Editor’s comments

1. *Thank you very much for submitting your manuscript "Pattern of COVID-19 in Sichuan Province, China: a descriptive epidemiological analysis" (#PONE-D-20-09556) for review by PLOS ONE. As with all papers submitted to the journal, your manuscript was fully evaluated by academic editor (myself) and by independent peer reviewers. The reviewers appreciated the attention to an important health topic, but they raised substantial concerns about the paper that must be addressed before this manuscript can be accurately assessed for meeting the PLOS ONE criteria. Therefore, if you feel these issues can be adequately addressed, we invite you to submit a revised version of the manuscript that addresses the points raised during the review process. We can’t, of course, promise publication at that time.*

- Thank you for offering us an opportunity to revise the manuscript. We have carefully addressed the comments raised by the reviewers and revised the manuscript accordingly. Please refer to the revised text for more details.

2. *If you would like to make changes to your financial disclosure, please include your updated statement in your cover letter.*

- Thank you. We have updated funds in the cover letter.

3. *To enhance the reproducibility of your results, we recommend that if applicable you deposit your laboratory protocols in protocols.io, where a protocol can be assigned its own identifier (DOI) such that it can be cited independently in the future. For instructions see: http://journals.plos.org/plosone/s/submission-guidelines#loc-laboratory-protocols*

- Thank you for your kindness. Our study is a descriptive epidemiological analysis and does not involve any laboratory protocols. we have fully disclosed our data in the supporting information.

4. *Please ensure that your manuscript meets PLOS ONE's style requirements, including those for file naming. The PLOS ONE style templates can be found at http://www.plosone.org/attachments/PLOSOne_formatting_sample_main_body.pdf and http://www.plosone.org/attachments/PLOSOne_formatting_sample_title_authors_affiliations.pdf.*

*Please ensure that your manuscript meets PLOS ONE's style requirements, including those for file naming.*

- We carefully reviewed the format requirements of *PLOS ONE* and revised inappropriate content according to the outline. We hope that the revised manuscript comply with the publishing requirements of *PLOS ONE*. If there is anything inappropriate, please point it out. We are happy to revise the manuscript according to the requirements of the editors.

5. *In the Methods, please provide a link to the official websites of the Health Commission in Sichuan, and/or describe how others may gain access to the data analysed in this study.*

*In addition, in the Methods, please state whether all patient data were fully anonymized before you accessed them.*

- We have updated the relevant information in the materials section of the manuscript, which can be find at L57-L77.

As follows,

All patient data were fully anonymized before we accessed them. All the patients data used in this study were obtained from the official websites of the 21 districts Health Commission in Sichuan province available at: http://cdwjw.chengdu.gov.cn/; http://www.zg.gov.cn/web/swsjsw; http://wjw.panzhihua.gov.cn/; http://wjw.luzhou.gov.cn/; https://wjw.deyang.gov.cn/; http://wjw.my.gov.cn/; http://wsjsw.cngy.gov.cn/; http://swjw.suining.gov.cn/; http://wsj.neijiang.gov.cn/; http://swjj.leshan.gov.cn/swjj/index.shtml; http://wsjsw.nanchong.gov.cn/; http://ybwjw.yibin.gov.cn/; http://wjw.guang-an.gov.cn/; http://wjw.dazhou.gov.cn/; http://wsjkw.cnbz.gov.cn/index.html; http://wjw.yaan.gov.cn/; http://swjw.ms.gov.cn/; http://swjw.ziyang.gov.cn/; http://wjw.abazhou.gov.cn/; http://wjw.gzz.gov.cn/; http://www.lsz.gov.cn/ztzl/rdzt/yqfk/yqtb/.

6. *Thank you for stating the following financial disclosure:*

*"The funders had no role in study design, data collection and analysis, decision to publish, or preparation of the manuscript."*

*At this time, please address the following queries:*

*a)	Please clarify the sources of funding (financial or material support) for your study. List the grants or organizations that supported your study, including funding received from your institution.*

*b)	State what role the funders took in the study. If the funders had no role in your study, please state: “The funders had no role in study design, data collection and analysis, decision to publish, or preparation of the manuscript.”*

*c)	If any authors received a salary from any of your funders, please state which authors and which funders.*

*d)	If you did not receive any funding for this study, please state: “The authors received no specific funding for this work.”*

*Please include your amended statements within your cover letter; we will change the online submission form on your behalf.*

- We have updated this information in our cover letter. This work was supported by the National Major Project of Science and Technology (No. 2017ZX10205501), Industrial Cluster Collaborative Innovation Project of Chengdu Technology Bureau (2016-XT00-00033-GX) and Sichuan Science and Technology Innovation (Miaozi Project) cultivation Project in 2019049 (2019). The funders had no role in study design, data collection and analysis, decision to publish, or preparation of the manuscript. No authors received a salary from any of our funders. Thanks for your kindness.

**Responses to the Reviewer #1**

*1. Has the statistical analysis been performed appropriately and rigorously?*

*Reviewer #1: I Don't Know*

- Thanks to the reviewers for the kind work on our manuscript. The results of statistical analysis have been included in the S1 File. Categorical variables were performed as percentages. Continuous variables were displayed as medians and interquartile ranges or simple ranges. We also show the results of statistical analysis in the manuscript.

2. *Have the authors made all data underlying the findings in their manuscript fully available?*

*Reviewer #1: No*

- All data had been stored in the S1 File, including the number of single-day diagnosis, demography (gender, age), regional distribution, incubation period, exposure history and symptoms.

3. Review Comments to the Author

*Please use the space provided to explain your answers to the questions above. You may also include additional comments for the author, including concerns about dual publication, research ethics, or publication ethics. (Please upload your review as an attachment if it exceeds 20,000 characters)*

*Reviewer #1: The study gives a descriptive analysis of 406 patients with SARS-CoV-2 infection in Sichuan province, China. I think the study can be accepted for publication because it gives some additional data on the COVID-19 in China. However, I am surprised to see very long incubation periods (median 12.5 days) among 40 patients who recalled their dates of exposure. For me, it is difficult to believe, because there are quite consistent estimates existent in the literature of about 5 days as the median. I suspect there is something wrong with the recalled dates: maybe the dates were with some uncertainty and the authors didn't account for this in their analysis. Maybe the selection of those 40 patients had some bias and maybe some of them were exposed later in time (~multiple exposure). I would still not follow the main recommendation of the authors to extend the quarantine length of 14 days to 28 days. Because these issues are not well-addressed in the manuscript, and there are no details on selected/excluded patients, I don't think that the present study will be accepted in the research community.*

- Thanks again to the reviewers for the contribution to our manuscript. We re-evaluated 487 cases and developed inclusion and exclusion criteria. 94 cases were included to explore the incubation period.

On the one hand, for the cases of living in or visiting Hubei, we defined the incubation period as the interval between the lastest departure from Hubei and earliest symptom onset. Because these cases were not recorded the date of exposure to the SARS-CoV-2. On the other hand, for the cases of close contact history, the incubation period was defined as the interval between the potential earliest date of contact of the transmission source (person with confirmed cases) and the potential earliest date of symptom onset (i.e., cough, fever, fatigue, or myalgia), which according to a literature from NEJM by Wei-jie Guan, Ph.D. et al(“Clinical Characteristics of Coronavirus Disease 2019 in China”). We excluded: 1) the cases that did not report the date of exposure to the SARS-CoV-2 and symptom onset. 2) patients with asymptomatic infection (it can be found on the L87-L96).

Finally, 94 cases were included and we found the median incubation period was 7 days ,ranging from 1 to 19 days (95% CI, 6.9 to 8.7 days), which within the scope of current literature report.

Thanks again to the reviewers. We are happy to revise it according to the opinions of the reviewers.

4. *L157: "This study tried to explore the incubation period of coronavirus cases." the verb "tried" is not good for using in scientific literature. I advice just to write something like "This study explored..."*

- We have revised it in the manuscript on the suggestion of the reviewer.

L104: “This study explored the incubation period of 94 patients”.

5. *L87: What exactly are those 21 official websites? Are those from subprovinces/cities of Sichuan province? It would good to specify it.*

- 21 official websites come from 21 districts (including 18 cities, 3 autonomous Prefecture) health commissions in Sichuan Province, which recorded COVID-19 cases each day. We have pointed this out in the manuscript.

L27-L28: Epidemiological information of 487 COVID-19 cases were collected from the official websites of 21 districts (including 18 cities, 3 autonomous prefecture) health commissions within Sichuan between 21st of January 2020 to 17th of April 2020.

L70-L72: We collected 487 confirmed COVID-19 cases on the official websites of the health committees of 21 districts in Sichuan province from 21^st^ of January 2020 to 17^th^ of April 2020.
