# Supplementary material for: Pattern of COVID-19 in Sichuan province, China: A descriptive epidemiological analysis
Source: PLoS One. 2020 Nov 5;15(11):e0241470. doi: 10.1371/journal.pone.0241470 (PMC7644260; doi:10.1371/journal.pone.0241470)
Supplement: Attachment — Submitted filename: Response to Reviewers.docx [file pone.0241470.s003.docx]

Dear Dr. Samy and Reviewers:

Thank you for your kind help in handling our manuscript ( #PONE-D-20-09556, Pattern of COVID-19 in Sichuan Province, China: a descriptive epidemiological analysis) to PLOS ONE.

We have performed the suggested analyses and revised the manuscript according to your editorial comments and the reviewers’ comments. We marked all new changes using "Track changes while editing" in the revised text. In details, we address the issues in the comments point-by-point as follows.

We hope that now, after the changes we have carried out in the light of the reviewers’ comments, our revised manuscript is in an appropriate format for publication in PLOS ONE. We look forward to hearing from you.

Thank you and best regards.

Yours sincerely,

Corresponding author: QuanshengFeng: School of Basic Medical Sciences, Chengdu University of Traditional Chinese Medicine,No.1166 Liutai Avenue,Wenjiang District, Chengdu, Sichuan, China 611137;

E-mail:fengqs118@163.com.

Editor’s comments

1. *Thank you very much for submitting your manuscript "Pattern of COVID-19 in Sichuan Province, China: a descriptive epidemiological analysis" (#PONE-D-20-09556R1) for review by PLOS ONE. As with all papers submitted to the journal, your manuscript was fully evaluated by academic editor (myself) and by independent peer reviewers. The reviewers appreciated the attention to an important health topic, but they raised substantial concerns about the paper that must be addressed before this manuscript can be accurately assessed for meeting the PLOS ONE criteria. Therefore, if you feel these issues can be adequately addressed, we invite you to submit a revised version of the manuscript that addresses the points raised during the review process. We can’t, of course, promise publication at that time.*

- Thank you for offering us an opportunity to revise the manuscript. We have carefully revised the manuscript according to the opinions of the reviewers. Please refer to the revised text for more details.

2. *If you would like to make changes to your financial disclosure, please include your updated statement in your cover letter.*

- Thank you. We do not have any *financial disclosure* to update.

3. *If applicable, we recommend that you deposit your laboratory protocols in protocols.io to enhance the reproducibility of your results. Protocols.io assigns your protocol its own identifier (DOI) so that it can be cited independently in the future. For instructions see: [http://journals.plos.org/plosone/s/submission-guidelines#loc-laboratory-protocols](http://journals.plos.org/plosone/s/submission-guidelines#loc-laboratory-protocols)*

- Thank you for your kindness. Our study is a descriptive epidemiological analysis and does not involve any laboratory protocols. we have fully disclosed our data in the supporting information.

**Responses to the Reviewer**

1. *If the authors have adequately addressed your comments raised in a previous round of review and you feel that this manuscript is now acceptable for publication, you may indicate that here to bypass the “Comments to the Author” section, enter your conflict of interest statement in the “Confidential to Editor” section, and submit your "Accept" recommendation.*

*Reviewer #1: (No Response)*

- Thanks to the reviewers. We feel sorry that the previous modification did not meet the PLOS ONE criteria. Now, we revise it again in accordance with the reviewers’ comments. We hope that this manuscript can meet the criteria of PLOS ONE.

2. Is the manuscript technically sound, and do the data support the conclusions?

   The manuscript must describe a technically sound piece of scientific research with data that supports the conclusions. Experiments must have been conducted rigorously, with appropriate controls, replication, and sample sizes. The conclusions must be drawn appropriately based on the data presented.

Reviewer #1:Partly

- Thanks. We have re-evaluated the data and found that our existing data does not support the estimation of the incubation period. In the future, we will collect data on the incubation period in Sichuan Province for further analysis. At the same time, we also found that the number of symptom-related cases should be 256 instead of 258. We have revised and marked in red in the manuscript (Table 1).

3. Has the statistical analysis been performed appropriately and rigorously?

Reviewer #1: No

- Thanks. We have revised the statistical analysis in the manuscript, especially the incubation period. We have carefully reviewed the data and found that the available data do not fully support the estimation of the incubation period. After reanalyzing the data, we found that the median time from the first symptom to the diagnosis can be estimated. This is of great significance to observe the coping ability and medical level of a region to SARS-CoV-2. After collecting more exact time of exposure to the virus, we will specifically estimate the incubation period of confirmed cases of COVID-19 in Sichuan province.

4. Have the authors made all data underlying the findings in their manuscript fully available?

The [PLOS Data policy](http://www.plosone.org/static/policies.action#sharing) requires authors to make all data underlying the findings described in their manuscript fully available without restriction, with rare exception (please refer to the Data Availability Statement in the manuscript PDF file). The data should be provided as part of the manuscript or its supporting information, or deposited to a public repository. For example, in addition to summary statistics, the data points behind means, medians and variance measures should be available. If there are restrictions on publicly sharing data—e.g. participant privacy or use of data from a third party—those must be specified.

Reviewer #1:Yes

- Done

5. Is the manuscript presented in an intelligible fashion and written in standard English?

PLOS ONE does not copyedit accepted manuscripts, so the language in submitted articles must be clear, correct, and unambiguous. Any typographical or grammatical errors should be corrected at revision, so please note any specific errors here.

Reviewer #1: Yes

- Done

6. Review Comments to the Author

Please use the space provided to explain your answers to the questions above. You may also include additional comments for the author, including concerns about dual publication, research ethics, or publication ethics. (Please upload your review as an attachment if it exceeds 20,000 characters)

Reviewer #1: "On the one hand, for the cases of living in or visiting Hubei, we defined the incubation period as the interval between the lastest departure from Hubei and earliest symptom onset. Because these cases were not recorded the date of exposure to the SARS-CoV-2." - I am sorry but this is wrong practice. The authors cannot do it, because it is not the incubation period. Incubation period is the time period from exposure to the virus to the illness onset. It does not equal to the time period from the latest departure date from Hubei to the symptoms onset. What the authors say is that some records of the data are censored. There are statistical methods to adress this and the authors need to do it properly.

Because a part of the results is incorrect, I cannot recommend this manuscript for acceptance and additional analysis is required.

- Thanks again to the reviewers for the contribution to our manuscript. We admit that there is a mistake in the definition of incubation period. The incubation period should be the period from exposure to the virus to the onset of symptoms, not from leaving Hubei to the onset of symptoms. We have reviewed a large number of literatures about the estimation of incubation period and found that almost all the models for estimating the incubation period of COVID-19 come from the literature which wrote by Reich et al in 2009. We should also use this model to estimate the incubation period. However, after establishing the inclusion and exclusion criteria, we found that the sample size available for latency estimation was too small, only more than 100 cases (a total of 487 cases), and the time of exposure to the virus in many confirmed cases could not be obtained. The calculated results are not representative. After obtaining more data on confirmed cases of COVID-19 in Sichuan province, we will specifically estimate the incubation period.

In addition, in the re-evaluation of the data, we found that the median time from the onset of the first symptoms to the diagnosis of local cases was 4 days, and that of imported cases was 4.5 days. We have provided the specific data in the supporting material.
